# The Activity of K_V_11.1 Potassium Channel Modulates F-Actin Organization During Cell Migration of Pancreatic Ductal Adenocarcinoma Cells

**DOI:** 10.3390/cancers11020135

**Published:** 2019-01-23

**Authors:** Sagar Manoli, Stefano Coppola, Claudia Duranti, Matteo Lulli, Lara Magni, Nirmala Kuppalu, Nikolaj Nielsen, Thomas Schmidt, Albrecht Schwab, Andrea Becchetti, Annarosa Arcangeli

**Affiliations:** 1 Department of Experimental and Clinical Medicine, University of Florence, Viale GB Morgagni 50, 50134 Florence, Italy; sagar.manoli@gmail.com (S.M.); stefano.coppola87@gmail.com (S.C.); claudia.duranti@unifi.it (C.D.); lara.magni@stud.unifi.it (L.M.); kurani17@gmail.com (N.K.); 2 Physics of Life Processes, Huygens-Kamerlingh Onnes Laboratory, Leiden University, Niels Bohrweg 2, 2333 CA Leiden, The Netherlands; Schmidt@Physics.LeidenUniv.nl; 3 Department of Experimental Biochemical and Clinical Sciences, University of Firenze, Viale GB Morgagni 50, 50134 Firenze, Italy; matteo.lulli@unifi.it; 4 Institut für Physiologie II, Robert-Koch-Str. 27b, D-48149 Münster, Germany; nikolaj_nielsen@hotmail.com (N.N.); aschwab@uni-muenster.de (A.S.); 5 Department of Biotechnology and Biosciences, University of Milano-Bicocca, Piazza della Scienza 2, 20126 Milano, Italy; andrea.becchetti@unimib.it

**Keywords:** pancreatic cancer, hERG1, fibronectin, desmoplastic matrix, hypoxia, focal adhesions, stress fibers, filopodia, integrins, intracellular Ca^2+^ concentration

## Abstract

Cell migration exerts a pivotal role in tumor progression, underlying cell invasion and metastatic spread. The cell migratory program requires f-actin re-organization, generally coordinated with the assembly of focal adhesions. Ion channels are emerging actors in regulating cell migration, through different mechanisms. We studied the role of the voltage dependent potassium channel K_V_11.1 on cell migration of pancreatic ductal adenocarcinoma (PDAC) cells, focusing on its effects on f-actin organization and dynamics. Cells were cultured either on fibronectin (FN) or on a desmoplastic matrix (DM) with the addition of a conditioned medium produced by pancreatic stellate cells (PSC) maintained in hypoxia (Hypo-PSC-CM), to better mimic the PDAC microenvironment. K_V_11.1 was essential to maintain stress fibers in a less organized arrangement in cells cultured on FN. When PDAC cells were cultured on DM plus Hypo-PSC-CM, K_V_11.1 activity determined the organization of cortical f-actin into sparse and long filopodia, and allowed f-actin polymerization at a high speed. In both conditions, blocking K_V_11.1 impaired PDAC cell migration, and, on cells cultured onto FN, the effect was accompanied by a decrease of basal intracellular Ca^2+^ concentration. We conclude that K_V_11.1 is implicated in sustaining pro-metastatic signals in pancreatic cancer, through a reorganization of f-actin in stress fibers and a modulation of filopodia formation and dynamics.

## 1. Introduction

Cell migration is a central feature of many physiological processes such as embryonic development, organ differentiation after birth, and the function of immune cells [1,2]. Conversely, several pathophysiological processes, such as wound healing and cancer invasion and metastasis, represent the downside of cell migration [3]. Cells are triggered to start a motility program by extracellular stimuli, in the form of either soluble molecules or adhesive interactions with the extracellular matrix (ECM). Such stimuli produce cyclical changes in cell adhesion and morphology, which overall underlie crawling motility [4]. Changes in cell adhesion occur through coordinated steps, which first comprise the occurrence of plasma membrane protrusion at the leading edge, followed by the formation of new adhesion sites under the protrusion; the disruption of older adhesion sites at the cell rear then occurs, to be finally followed by contraction, which hence results in cell body movement [4]. Changes in cell morphology are driven by the constant remodeling of filamentous (f)-actin at specific sites, into structures (i.e., filopodia, lamellipodia, and stress fibers) that coordinate cell migration. They are regulated by different, specific signaling pathways [5,6]. The lamellipodium is characterized by a dense network of short, branched actin filaments, driven by activation of the Arp2/3 complex [7,8], while filopodia are transient, thin, hairlike protrusions that contain parallel actin bundles, whose polymerization is apparently driven by members of the formin family [9,10]. Stress fibers are long actin filaments linked by α-actinin and myosin, whose contraction enables forward movement of the cell body [11]. Indeed, stress fibers are physically linked to focal adhesions to allow mechanical integration of actin and adhesion dynamics during cell migration [11]. Rho GTPases bind to a variety of effectors, including protein kinases and some actin-binding proteins. These directly or indirectly affect the local assembly or disassembly of filamentous (f)-actin [12]. Overall, the local assembly or disassembly of f-actin represent central hubs of cell motility and migration [13].

Another component of the migratory machinery, largely neglected until quite recently, is represented by ion transport proteins (ion channels and transporters) across the plasma membrane. Ion channels and transporters regulate cell migration through different mechanisms: Setting the cell membrane potential, regulating cell volume, modulating intra- and/or extra-cellular pH and, most importantly, controlling intracellular Ca^2+^ concentration ([Ca^2+^]_i_) [14]. For example, the local swelling facilitates the outgrowth of protrusions at the leading edge while local shrinkage accompanies the retraction of the cell rear. Cytosolic Ca^2+^ concentrations exert their main effects on cytoskeletal dynamics via motor proteins such as myosin or dynein [15]. The fine-tuned control of [Ca^2+^]_i_ is therefore one of the main determinants underlying cell motility programs. It is therefore not surprising that different types of Ca^2+^ channels, both voltage-dependent and voltage-independent such as TRP channels, as well as those channels, mainly K^+^ channels, which regulate Ca^2+^ homeostasis through the resting potential (Vm) and hence the driving force for Ca^2+^, are functionally linked to the motility machinery [15,16]. In addition to the actual process of ion transport, channels, and transporters contribute to cell migration by being part of focal adhesion complexes and/or physically interacting with cell adhesion receptors, in particular integrins as well as with components of the cytoskeleton [17]. For example, f-actin has been shown to interact with as many as 10 different ion channels. Various biochemical and biophysical assays reveal that f-actin and ion channels are connected directly or through adaptor proteins [18]. Notably, inside these complexes ion channels can exert their effects on cell migration also via non-conductive mechanism, through the functional modulation of associated proteins [19,20].

One of the ion channels mainly involved in regulating intracellular signaling pathways triggered by cell adhesion to the ECM in several types of cancer cells is the voltage dependent potassium channel K_V_11.1 (also named human ether-à-go-go-related gene 1, hERG1) [21,22]. K_V_11.1 activity can activate pro-migratory programs in different types of neoplastic cells [23,24,25], through a functional cross talk with β1-containing integrin receptors, which, once activated by ECM proteins such as Fibronectin (FN), activate K_V_11.1 channels, which in turn modulate integrin-dependent intracellular signaling pathways [26].

Based on these premises, the aim of the present study was to define the mechanisms underlying the pro-migratory effects of K_V_11.1 channel activity focusing on the organization and dynamics of the actin cytoskeleton. We addressed this point in PDAC cells, since PDAC is characterized by a peculiar desmoplastic and hypoxic tumor microenvironment that triggers a pro-migratory program which accounts for the highly malignant and invasive behavior of this cancer.

## 2. Results

### 2.1. F-Actin Organization and Migratory Activity of PANC-1, PDAC Cells Cultured on Fibronectin (FN): Role of K_V_11.1 Channels

**Figure 1:**
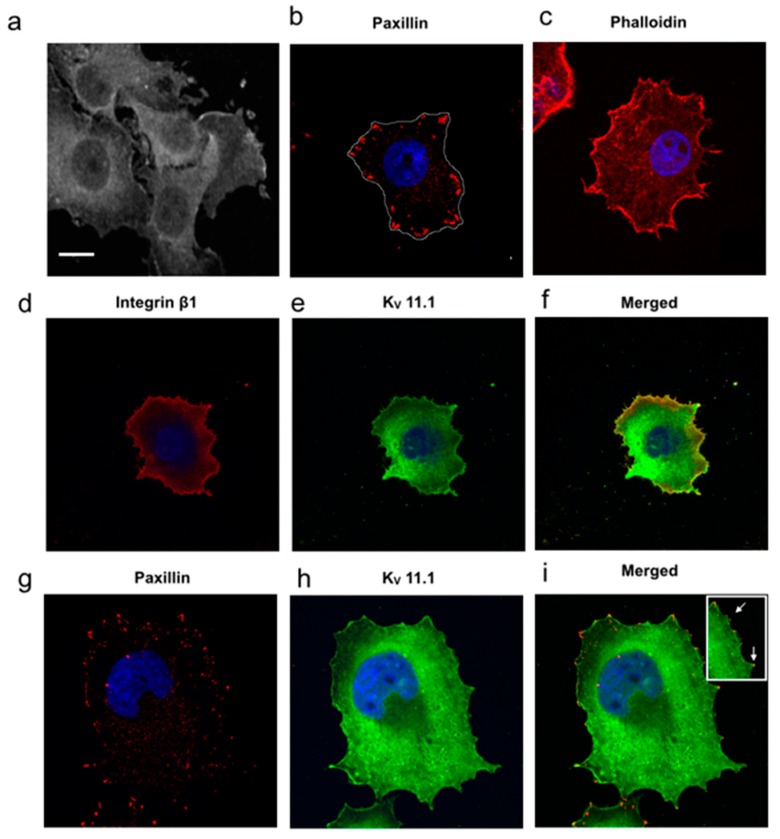
Phalloidin staining, K_V_11.1, β1 integrin and paxillin expression in PANC-1, Pancreatic Ductal Adenocarcinoma cells cultured onto fibronectin (FN). (**a**) Representative confocal image of spread and adherent PANC-1 cells. Scale bar: 10 μm. Scale bar is the same for all the subpictures. (**b**) Representative confocal single cell image of fixed PANC-1 cells stained with anti-paxillin primary antibody and revealed with AF546-conjugated anti-mouse secondary antibody. Nuclei are Hoechst stained in blue. (**c**) Representative single cell image of fixed PANC-1 cells. Actin staining by Rhodamine-conjugated phalloidin (red). (**d**–**f**) Co-localization of K_V_11.1 and β1 integrin. Cells were stained for β1 integrin with ts2/16 primary antibody and revealed with AF546-conjugated anti-mouse secondary antibody; for K_V_11.1, anti-hERG1 mAb-AF488 conjugated antibody. Co-localization of K_V_11.1 and β1 integrin is reported (Merged, third panel from the left). (**g**–**i**) Co-localization of K_V_11.1 and paxillin. Cells were stained with anti-paxillin primary antibody and revealed with AF546-conjugated anti-mouse secondary antibody (**g**); for K_V_11.1, cells were stained using anti-hERG1 mAb-AF488 conjugated antibody (**h**). Merged image (**i**) shows that co-localization of Paxillin and K_V_11.1 mainly occurs, as better evident in the inset with a higher magnification (scale bar missing for inset) showing the overlay of K_V_11.1 and paxillin staining.

To define the mechanisms underlying the pro-migratory role of K_V_11.1 in PDAC cells, PANC-1 cells were cultured in two different experimental conditions: (1) on FN in the presence of serum-free, BSA-containing medium; (2) on a collagen-rich “desmoplastic” matrix (DM) in the presence of a serum free-medium conditioned by activated human pancreatic stellate cells (PSCs), cultured in hypoxia (Hypo-PSC-CM). After six hours of incubation onto FN PANC-1 cells appeared firmly adherent and spread onto the substrate (Figure 1a), with evident focal adhesions, witnessed by Paxillin staining (Figure 1b). Under these conditions, intracellular f-actin, stained by Rhodamine-conjugated phalloidin, was organized as intracellular and cortical stress fibers (Figure 1c). β1 integrins (Figure 1d) and K_V_11.1 channels (Figure 1e) were both expressed, with spots of co-localization, as expected [25] (Figure 1f). The co-localization of β1 and K_V_11.1 is corroborated by the co-immunoprecipitation between the two proteins (see Figure 4, panel g). K_V_11.1 was present in focal adhesions, in conjunction with paxillin (Figure 1g–i).

**Figure 2:**
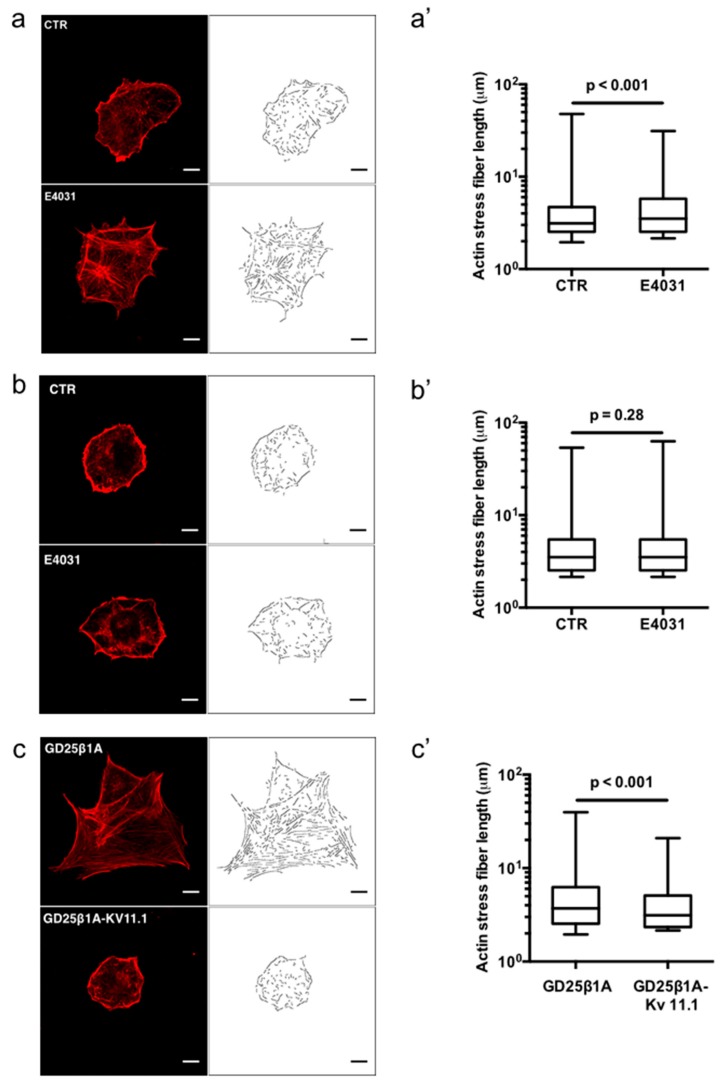
Actin stress fiber formation in PANC-1, BxPC3, and GD25 cells cultured onto FN. (**a**) Representative confocal images of fixed PANC-1 cells in the absence (control (CTR)) and presence of 40 μM E4031 (E4031). Actin staining by rhodamine-conjugated phalloidin (red). The right panels show the detected and segmented stress fibers from the actin signal (see Materials and Methods section for details). Scale bars: 10 μm. (**a’**) Distribution of actin stress fibers in PANC-1 cells in CTR and E4031 conditions. Boxes include central 50% of data points, the horizontal lines denote minimum value, median and maximum value. At least a total of 40 cells per condition from three independent experiments were analyzed. All *p*-values were determined by a Mann–Whitney test (significant level set to *p* < 0.05), or for data deviating from normality by a Kolmogorov–Smirnov test. (**b**) Representative confocal images of fixed BxPC3 cells in the absence (CTR) and presence of 40 μM E4031 (E4031). Actin staining by Rhodamine-conjugated phalloidin (red). The right panels show the detected and segmented stress fibers from the actin signal (see Materials and Methods section for details). Scale bars: 10 μm. (**b’**) Distribution of actin stress fibers in BxPC3 cells in CTR and E4031 conditions. Boxes include central 50% of data points, the horizontal lines denote minimum value, median and maximum value. At least a total of 40 cells per condition from three independent experiments were analyzed. All *p*-values were determined by a Mann–Whitney test (significant level set to *p* < 0.05), or for data deviating from normality by a Kolmogorov–Smirnov test. (**c**) Representative single cell image of GD25β1A and GD25β1A-K_V_11.1 cells. Actin staining by rhodamine-conjugated phalloidin (red). The right panels show the detected and segmented stress fibers from the actin signal (see Materials and Methods section for details). Scale bars: 10 μm. (**c’**) Distribution of actin stress fibers in GD25β1A and GD25β1A-K_V_11.1 cells. Boxes include central 50% of data points, the horizontal lines denote minimum value, median and maximum value. At least a total of 40 cells per condition from three independent experiments were analyzed. All *p*-values were determined by a Mann–Whitney test (significant level set to *p* < 0.05), or for data deviating from normality by a Kolmogorov–Smirnov test.

The role of K_V_11.1 in f-actin organization was then studied by determining the effects of the K_V_11.1 specific blocker E4031, used at a concentration (40 µM) proven to be effective to block K_V_11.1 currents in a rich, protein-containing medium [27]. E4031 produced a change in the organization of stress fibers, which appeared significantly longer compared to control (CTR) untreated cells (median values 3.5 and 3.0 µm, respectively, *p* < 0.001) (Figure 2a,a’). E4031 did not exert any effect on stress fibers’ length of another PDAC cell line, BxPC3 (median values 3.5 and 3.5 µm, respectively, *p* = 0.28) which barely express K_V_11.1 [25], and show stress fibers significantly longer than PANC-1 cells (median values 3.5 and 3.0 µm, respectively, *p* < 0.001) (Figure 2b,b’). These data suggest that K_V_11.1 contributes to keep f-actin in a less organized arrangement in stress fibers of PANC-1 cells. This conclusion was corroborated studying GD25β1A cells (i.e., mouse cells knocked out for β1, in which the human β1A integrin was transfected) in which K_V_11.1 channels were exogenously expressed, GD25β1A-K_V_11.1. GD25β1A-K_V_11.1 cells show less organized stress fibers, with shorter f-actin filaments compared to native GD25β1A cells (median values 3.1 and 3.7 µm, respectively, *p* < 0.001) (Figure 2c,c’). A similar effect was observed in HEK 293 cells transfected with K_V_11.1 (Figure S1); similarly, not-transfected GD25β1A and HEK cells behaved alike.

**Figure 3:**
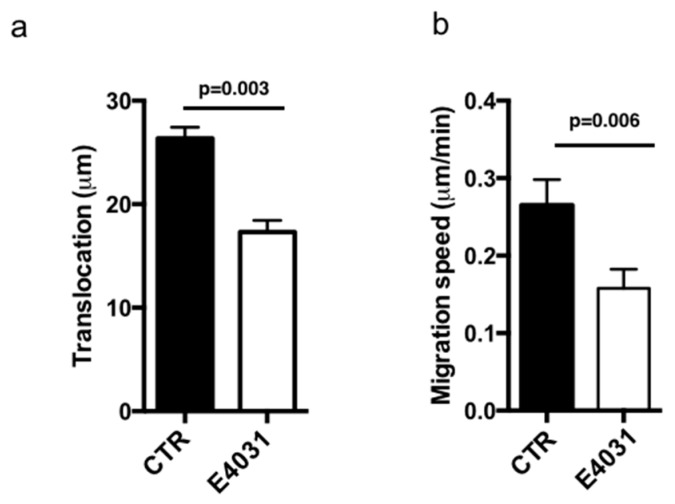
Cell migration of PANC-1 cells cultured onto FN. (**a**) Translocation data. Cells were incubated on FN in the absence (CTR) or in the presence of 40 μM E4031 (E4031). Images of at least 40 cells acquired during 4 h time lapse were analyzed using ImageJ and it was calculated as the net distance between the starting and the end position of the cell. Data are reported as mean ± SEM. All *p*-values were determined by a Mann–Whitney test (significant level set to *p* < 0.05), or for data deviating from normality by a Kolmogorov–Smirnov test. (**b**) Migration speed data. Cells were incubated on FN in the absence (CTR) or in the presence of 40 μM E4031 (E4031). Images of at least 40 cells acquired during 4 h time lapse were analyzed using ImageJ. Migration speed was calculated as the distance travelled by the cell center per min. Data are reported as mean ± SEM. All *p*-values were determined by a Mann–Whitney test (significant level set to *p* < 0.05), or for data deviating from normality by a Kolmogorov–Smirnov test.

We then studied the role of K_V_11.1 on cell migration of PANC-1 cells, seeding them onto FN for two hours and collecting time-lapse images for further four hours in the absence or in the presence of E4031. Single cell traces were analyzed and both translocation (net distance covered during the course of the experiment; measured in µm) and migration speed (measured as µm/min) were determined. The treatment with E4031 produced a statistically significant decrease in both parameters (Figure 3a,b).

Overall, the effect of K_V_11.1 in PDAC cells cultured on FN is that of lowering stress fibers’ length, maintaining them short enough to sustain the pushing force necessary for cell migration to occur. Blocking K_V_11.1 activity result in an elongation of stress fibers and a subsequent impairment in cell migration.

### 2.2. F-Actin Organization and Migratory Activity of PANC-1 PDAC Cells Cultured on a Desmoplastic Microenvironment: Role of K_V_11.1 Channels

**Figure 4:**
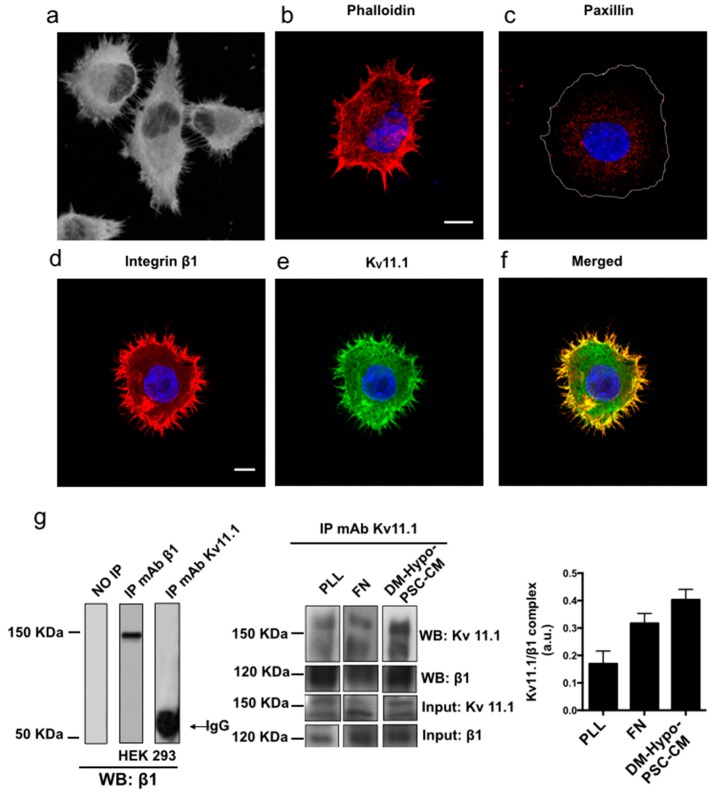
Phalloidin staining, K_V_11.1, β1 integrin and paxillin expression in PANC-1 cells cultured onto desmoplastic matrix (DM) plus hypoxia (Hypo-PSC-CM). (**a**) Representative confocal image of PANC-1 cells. Scale bar: 10 μm. (**b**) Representative single cell image of fixed PANC-1 stained with anti-paxillin primary antibody and revealed with AF546-conjugated anti-mouse secondary antibody. Nuclei are Hoechst stained in blue. (**c**) Representative single cell image of fixed PANC-1. Actin staining by rhodamine-conjugated phalloidin (red). (**d**–**f**) Cells were stained for β1 integrin with ts2/16 primary antibody and revealed with AF546-conjugated anti-mouse secondary antibody; for K_V_11.1, anti-hERG1 mAb-AF488 conjugated antibody. Co-localization of K_V_11.1 and β1 integrin is reported (Merged, third panel from the left). (**g**) Co-immunoprecipitation of K_V_11.1 and β1 integrin in PANC-1 cells after seeding on Polylysine (PLL), FN, or DM plus Hypo-PSC-CM, for 90 min. Total lysate was immunoprecipitated using anti-hERG1 antibody (IP mAb K_V_11.1). Western blotting was performed on immunoprecipitates and total lysates (input) from the same sample, with either anti-K_V_11.1 antibody or anti-β1 antibody. The corresponding densitometric results are given in the bar graph on the right. The control data are shown in the left panel: left lane no immunoprecipitate; middle lane immunoprecipitate on HEK 293 cells with anti β1 antibody (molecular weight immunoprecipitated β1 integrin approximately 120 kDa); right lane immunoprecipitate on HEK 293 cells (not expressing K_V_11.1) with anti-hERG1 antibody (IP mAb K_V_11.1). Wester blotting was performed using anti β1 antibody.

The role of K_V_11.1 in cell migration and f-actin organization was then studied in PANC-1 PDAC cells cultured on a collagen-rich desmoplastic matrix plus Hypo-PSC-CM [28]. After six hours of culture under these conditions, PANC-1 cells were attached although less spread than on FN (Figure 4a). Numerous thin protrusions, clearly labeled with phalloidin, resembling filopodia, were evident at the cell periphery (Figure 4b).

On the contrary, stress fibers were barely detectable. Consistently, focal adhesions were rare and sparse throughout the cell, as witnessed by paxillin staining (Figure 4c). β1 integrins were mainly localized at the tips of such protrusions (Figure 4d), confirming their filopodial nature. Also K_V_11.1 was present in filopodia (Figure 3e), and co-localized with β1 integrins (Figure 4f). Interestingly, K_V_11.1 and β1 integrin co-immunoprecipitated in PANC-1 cells cultured in DM plus Hypo-PSC-CM at higher extent when compared to what occurred when cells were cultured on FN (Figure 4g). Overall, PANC-1 cells cultured on DM plus Hypo-PSC-CM emit numerous filopodia, and hence represent a good model wherein to study the role of K_V_11.1 on filopodia organization.

### 2.3. K_V_11.1 Activity Modulates F-Actin Organization and Dynamics in Filopodia of PANC-1, PDAC Cells Cultured on a Desmoplastic Matrix

**Figure 5:**
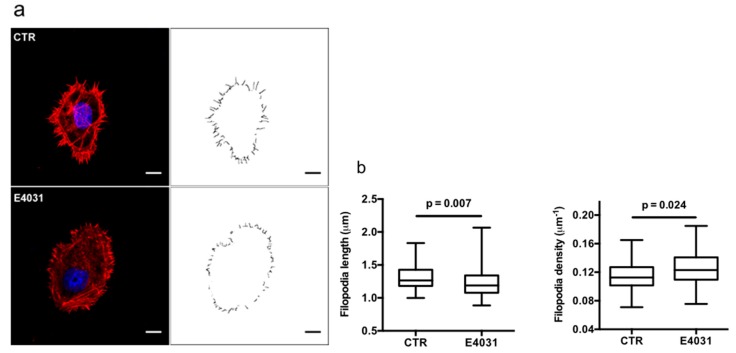
Actin filopodia formation in PANC-1 cells cultured onto DM plus Hypo-PSC-CM. (**a**) Representative confocal images of fixed PANC-1 cells in the absence (CTR) and presence of 40 μM E4031 (E4031), after Hypo-PSC-CM stimulation. Actin and nuclei staining by rhodamine-conjugated phalloidin (red) and DAPI (blue). The right panels show the detected and segmented filopodia from the actin signal (see Materials and Methods section for details). Scale bars: 10 μm. (**b**) Distribution of filopodia length averaged for each cell in CTR and E4031 conditions (left panel). Distribution of filopodia linear density calculated as the number of detected filopodia normalized for the cell perimeter in CTR and E4031 conditions (right panel). Boxes include central 50% of data points, the horizontal lines denote minimum value, median, and maximum value. At least a total of 40 cells per condition from three independent experiments were analyzed. All *p*-values were determined by a Mann–Whitney test (significant level set to *p* < 0.05), or for data deviating from normality by a Kolmogorov–Smirnov test.

The effect of K_V_11.1 on f-actin organization in filopodia was hence tested by analyzing the effects of E4031. Figure 5a shows representative confocal images of phalloidin-stained f-actin of PANC-1 cells in the absence or in the presence of E4031. Filopodia were detected and their length and density were measured (top and bottom right images in Figure 5a). When treated with E4031, PANC-1 cells displayed filopodia that were significantly shorter (median values 1.2 and 1.3 µm, respectively, *p* = 0.007), although with significantly higher density (median value 0.12 and 0.11 µm-1, respectively, *p* = 0.024) compared to control conditions, CTR (Figure 5b).

**Figure 6:**
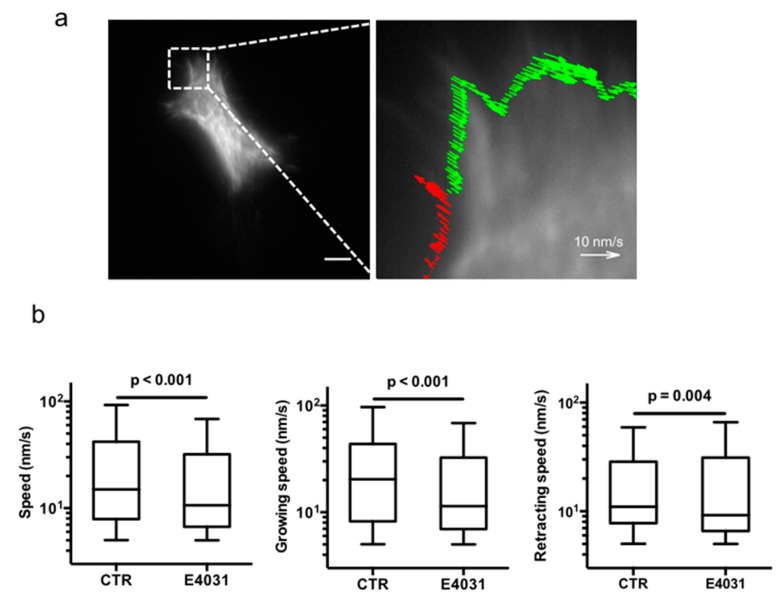
Actin dynamics in PANC-1 cells cultured onto DM plus Hypo-PSC-CM. (**a**) Representative total internal reflection fluorescence (TIRF) image of live GFP Life-Act transfected PANC-1 cells in control conditions (CTR), after Hypo-PSC-CM stimulation. (Scale bar: 10 μm). The time series (120 images at 1 Hz) were analyzed by STICS, to build local velocity maps within the cell perimeter, as shown in the enlargement (right panel). Red and green arrows indicate growing and retracting speed, respectively. (**b**) Distributions of filopodia polymerization speed (left panel), filopodia growing and retraction speed calculated as velocities pointing outward (middle panel) and inward (right panel) the cell perimeters for cells in CTR and in the presence of 40 μM E4031 (E4031). Boxes include central 50% of data points, the horizontal lines denote minimum value, median, and maximum value. At least 15 time series per condition from three independent experiments were analyzed. All *p*-values were determined by a Mann–Whitney test (significant level set to *p* < 0.05), or for data deviating from normality by a Kolmogorov–Smirnov test.

We then tested whether these effects could be traced back to a modulation of actin dynamics in filopodia. For this purpose, cells were first transfected with lifeAct-GFP and then GFP-labeled actin filaments were visualized by total internal reflection fluorescence (TIRF) microscopy in live cells, by acquiring 120 images at 1 Hz. To assess the actin polymerization speed, the time series of images were analyzed using spatio-temporal image correlation spectroscopy (STICS) [29]. Figure 6a shows a representative TIRF image of GFP-labeled actin, superimposed in the enlargement by the actin flow map (red and green arrows) calculated on the cell perimeter (i.e., restricted to filopodia) using STICS, as described in the Materials and Methods section. In agreement with the results on the filopodia length, the inhibitory effect on K_V_11.1 by E4031 caused a significant decrease in the actin polymerization speed (left panel, Figure 6b). Interestingly, both growing and retraction speed components were significantly reduced in E4031-treated cells, suggesting that K_V_11.1 affects analogously the assembly and disassembly of actin filopodia (middle and right panel, Figure 6b). Such mechanism is illustrated in Supplementary video (Movie S1).

**Figure 7:**
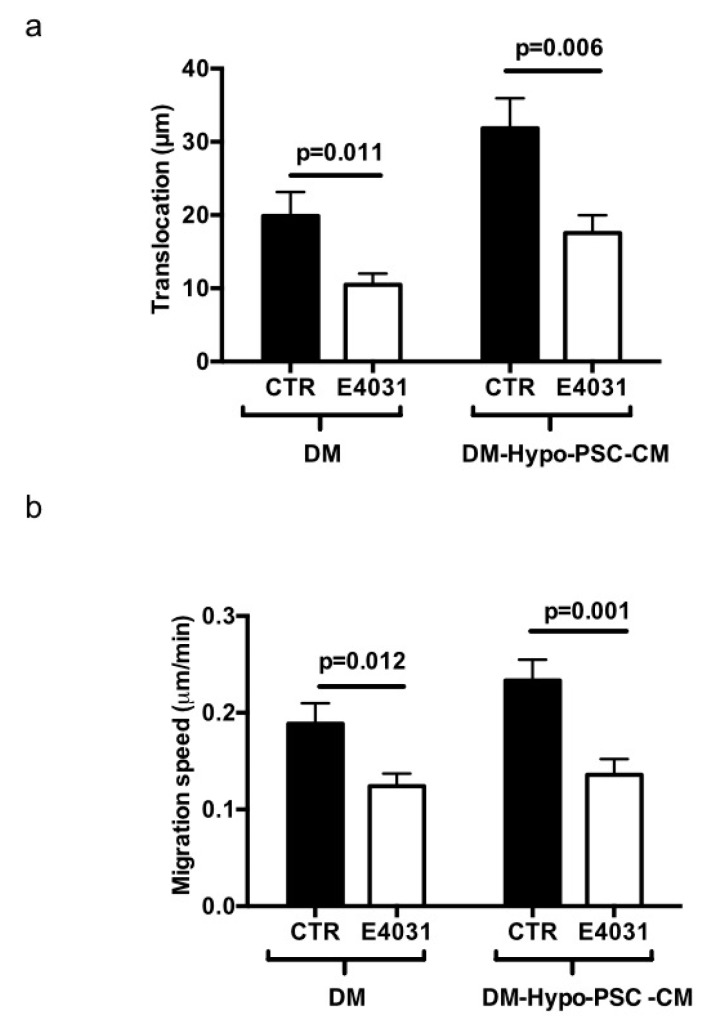
Cell migration in PANC-1 cells cultured onto DM and DM plus Hypo-PSC-CM. Cells were incubated for 24h on DM in the absence (non-conditioned medium, NCM) or in the presence of conditioned medium (human pancreatic stellate cells (PSCs)-conditioned medium, PSC-CM), in the absence (CTR) or in the presence of 40 μM E4031 (E4031). Conditioned medium was collected from PSCs grown in hypoxia (1% O_2_) (Hypo-PSC-CM). The translocation (**a**) and migration speed (**b**) are increased upon treatment with conditioned medium. This effect is largely reversed by K_V_11.1 channel inhibition. K_V_11.1 channel inhibition elicits larger effects in PANC-1 cells stimulated with hypoxic conditioned medium. Data are reported as mean ± SEM. All *p*-values were determined by a *t*-test (significant level set to *p* < 0.05), or for data deviating from normality by a Kolmogorov–Smirnov test.

We then quantified cell migration of PANC-1 cells under these conditions, applying the same procedure as described in Figure 2. When cultured on DM, PANC-1 cells showed a clear pro-migratory activity, which was further increased by the addition of Hypo-PSC-CM, as previously reported [28]. The addition of the K_V_11.1 specific blocker E4031 significantly reduced both cell migration speed and translocation (Figure 7a,b).

To summarize, K_V_11.1 channels exert a significant pro-migratory effect on PANC-1 cells cultured on DM and triggered to migrate by the addition of Hypo-PSC-CM, which is due to enhanced f-actin dynamics in filopodia.

### 2.4. Effects of K_V_11.1 Activity on Intracellular Ca^2+^ Concentrations of PANC-1 PDAC Cells Cultured on either FN or a Desmoplastic Microenvironment

**Figure 8:**
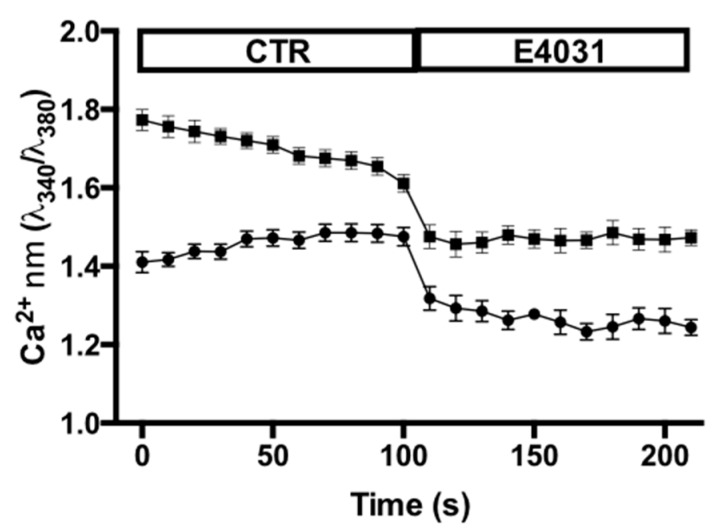
Effect of E4031 on intracellular Ca^2+^ in PANC-1 cells cultured onto FN (circles) or DM plus Hypo-PSC-CM (squares). [Ca^2+^]_i_ was determined by Fura2 fluorescence intensity as detailed in Materials and Methods in the absence (CTR) or in the presence of 40 µM E4031 (E4031). At least 25 cells per condition from 3 independent experiments were analyzed. Data are reported as Ca^2+^ ratio at λ_340_/λ_380_. Data are mean ± SEM. Average values of PANC-1 in control versus E4031 conditions on FN are significantly different: *p* < 0.0001. Average values of PANC-1 in control versus E4031 conditions on DM plus Hypo-PSC-CM are significantly different: *p* < 0.0001. Average values of PANC-1 in control conditions on FN versus DM plus Hypo-PSC-CM are significantly different: *p* < 0.0001. *p* values were determined by ANOVA test (significant level set to *p* < 0.05).

Finally, we analyzed whether the above effects of K_V_11.1 could be associated to a modulation of intracellular Ca^2+^ concentration ([Ca^2+^]_i_). To this purpose, PANC-1 cells were cultured either on FN or on DM plus Hypo-PSC-CM for 2hr and [Ca^2+^]_i_ was determined by Fura-2 imaging, as in [28]. After 10 min incubation, E4031 was applied and [Ca^2+^]_i_ was followed, acquiring pictures every 10s, for 10 min. Data relative to the last 2 min in control conditions (CTR) and after the application of E4031 are shown in Figure 8. E4031 significantly decreased intracellular Ca^2+^ of cells seeded either on FN or in DM plus Hypo-PSC-CM. In the latter condition, cells showed a slightly higher [Ca^2+^]_i_.

## 3. Discussion

Cell migration is a key process in cancer, as it underlies invasion and metastatic spread, which in turn determine tumor malignancy. Hence, defining the cellular and molecular mechanisms that trigger and sustain cell migration is crucial especially in those cancers, such as PDAC, whose malignancy mainly relies to the early occurrence of metastases, and thus to the activation of pro-migratory programs.

In the present paper, we provide evidence that the voltage dependent potassium channel K_V_11.1 promotes cell motility programs in PDAC cells, through a complex regulation of f-actin assembly and dynamics, both in stress fibers and in filopodia.

The central role of ion channels and transporters in the regulation of cell motility has clearly emerged in the last fifteen years [14]. This occurs thanks to the regulation of f-actin, which can be direct or mediated by the control of intracellular Ca^2+^ concentrations [15,16]. Besides Ca^2+^ channels [30], K^+^ channels are also implicated in modulating cell migratory programs, through different mechanisms, either on ion conduction or independent of ion flux (non-conductive) [20,22]. The latter mechanism can occur since several K^+^ channels can physically interact with important molecules of the cell migration apparatus, such as FAK [31], cortactin [32], and integrins [33,34]. For example, inwardly rectifying K_IR_4.2 channels can associate and co-localize with α9β1 integrins at the leading edge of the lamellipodium, hence promoting lamellipodia formation [35,36]. We had already provided evidence that K_V_11.1 channels mediate transendothelial migration in Acute Myeloid Leukemia cells [24], and promote cell migration through a basement membrane-like ECM in PDAC cells [25], since they modulate integrin-dependent intracellular signaling pathways [26].

**Figure 9:**
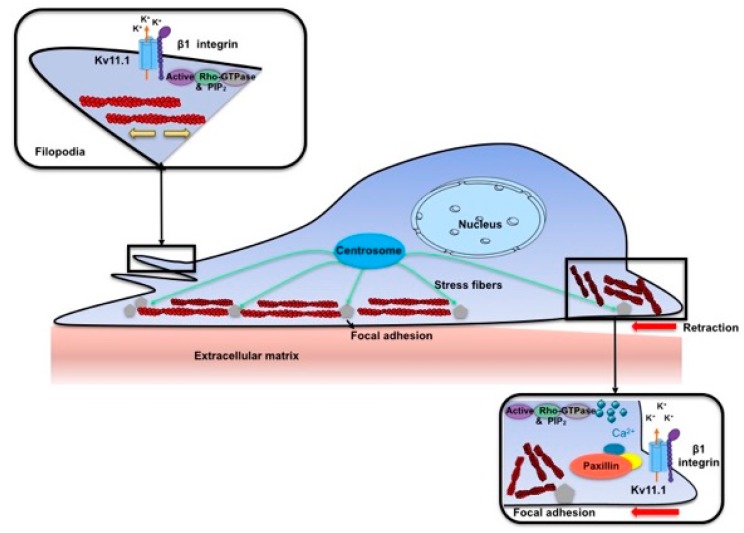
Proposed mechanism for the modulation of f-actin organization exerted by K_V_11.1 channels during cell migration of pancreatic ductal adenocarcinoma cells. The migratory machinery is a complex unit in pancreatic ductal adenocarcinoma (PDAC) cells and it is represented by the centrosome which directs focal adhesion and actin rearrangement. We propose that K_V_11.1 channels are essential to maintain stress fibers in a less organized format (as represented on the upper left side inset) in PANC-1 filopodia, while in focal adhesion (right lower panel) K_V_.11.1 complexes with β1 integrin modulating downstream signaling molecules, such as paxillin, impacting on actin organization which results to be more disorganized. We propose that this is a Ca^2+^ dependent mechanism.

We here studied in more details the molecular mechanisms underlying the role of K_V_11.1 in cancer cell motility, using PDAC cells as a model, in two experimental conditions: cells adhering and migrating onto a single ECM protein, FN, and cells cultured on a desmoplastic, collagen-rich matrix, with the addition of a conditioned medium produced by pancreatic stellate cells cultured in hypoxia (DM plus Hypo-PSC-CM), which better mimics the PDAC microenvironment. These two culture conditions allowed us to reveal that the two main types of f-actin organization were differentially affected by the activity of K_V_11.1 channels. In fact, PDAC cells cultured on FN present high levels of focal adhesion and an intracellular f-actin mesh organized into stress fibers, thus mimicking the subnuclear and rear front of a migrating cell. In contrast, cells cultured on DM plus Hypo-PSC-CM are characterized by the emission of numerous, thin filopodia. The latter are structures that migrating cells use to probe substrate rigidity, before spreading of lamellipodia [37]. K_V_11.1 produced a complex re-organization of f-actin, contributing to maintain stress fibers in a less organized format in cells cultured on FN, whereas it promoted the organization of cortical f-actin into sparse and long filopodia, and allowed quick f-actin polymerization, in cells cultured on DM plus Hypo-PSC-CM (Figure 9). In the latter condition, both growing and retraction speed components were significantly reduced in cells in which the activity of K_V_11.1 channels was blocked, suggesting that K_V_11.1 similarly affects the assembly and disassembly of actin filopodia.

The two different substrates we used, FN versus DM, can produce different organization of f-actin presumably because of their different rigidity (the latter being softer), and spatial organization, 2D and 3D, respectively. On rigid matrices, which cause a default cell response of active extension/spreading, focal adhesions mature thanks to the maintenance of GTPase Rac and Arp 2/3-mediated actin polymerization [12,13]. Soft substrates, on the other hand, especially in 3D, determine tension at nascent adhesions, avoiding their maturation into focal adhesions and promoting the retraction of filopodia. In softer, more complex, 3D environments, cells cannot form lamellipodia or focal adhesions but are still capable to form other types of extensions, e.g., filopodia [37]. Moreover, while we did not add any soluble factor to cells cultured onto FN, the Hypo-PSC-CM might contain several growth factors/cytokines released by activated PSC, capable to further modulate the organization of f-actin, as well as [Ca^2+^]_i_ In fact, cells cultured onto DM plus Hypo-PSC-CM had a slightly higher [Ca^2+^]_i_, compared to those cultured onto FN (Figure 8). This difference may depend on the presence, in Hypo-PSC-CM, of factors known to trigger Ca^2+^ release from intracellular stores, e.g., PDGF, thus leading to an increased intracellular Ca^2+^.

How can K_V_11.1 modulate f-actin arrangements and dynamics in stress fibers or filopodia? One possibility is that K_V_11.1 activity modulates [Ca^2+^]_i_, through the regulation of the cell resting potential, which affects the driving force for Ca^2+^. This would imply the involvement of plasma membrane Ca^2+^ channels, in addition to those mediating Ca^2+^ release from intracellular stores. Indeed, E4031 significantly decreased [Ca^2+^]_i_ in cells cultured onto either FN or DM plus Hypo-PSC-CM, and the effect started very quickly after the drug was added. Furthermore, K_V_11.1 is known to form a signaling complex with the β1 subunit of integrin receptors. We confirmed that K_V_11.1 co-localizes and co-immunoprecipitates with β1 integrin in PDAC cells, in both the culture conditions used in the present paper. Interestingly, K_V_11.1 mainly localizes to focal adhesions in cells cultured on FN and in filopodia when cells are cultured on DM plus Hypo. In both conditions, K_V_11.1 is adjacent, or even complexed with the β1 integrin. Moreover, we previously demonstrated that K_V_11.1 co-immunoprecipitates with Rho GTPases, and its activity regulates Rac [26]. Hence, our present working hypothesis is that this mechanism could mediate the effect of K_V_11.1 on f-actin organization in regulating filopodia remodeling.

Finally, since f-actin is able to bind several ion channel types [18], and K_V_11.1 directly interacts with the actin binding protein TRIOBP1 in cardiac cells [38], it is conceivable that K_V_11.1 directly affects f-actin organization and dynamics. Interestingly, the latter mechanisms imply that K_V_11.1 can modulate cell migration, and hence cancer metastasis, also by non-conductive mechanisms, in analogy with what was shown in breast cancer cells [20,22].

## 4. Materials and Methods

### 4.1. Cell Culture

The PDAC cell line PANC-1 and BxPc3 were cultured in Dulbecco’s Modified Eagle Medium (DMEM) freshly supplemented with 4 mM l-glutamine and 10% fetal bovine serum (FBS), and cultured at 37 °C and 5% CO_2_ unless mentioned otherwise. Immortalized PSCs were cultured in DMEM-F12 media pre-supplemented with l-glutamine and 10% FBS. For hypoxia stimulation or activation, semi-to-fully confluent PSCs were incubated at 37 °C in serum-free DMEM media in the hypoxic incubator (1% O_2_, (Concept 400, Jouan, Milan, Italy)) for not more than 18 h.

### 4.2. Desmoplastic Matrix (DM) Coating

Culture plates were coated with a cocktail of five extracellular matrix (ECM) proteins, as reported in [27]. Collagen-I (800 μg/mL, Millipore), collagen-III (12 μg/mL, BD Bioscience San Jose, CA, USA, collagen-IV (5.4 μg/mL, BD Bioscience), fibronectin (40 μg/mL, Sigma Aldrich) and laminin (40 μg/mL, Sigma Aldrich, St. Louis, MO, USA and DMEM (10.4 g/L, Euroclone), HEPES (10 mM), NaOH (4.8 mM) were prepared and used for coating dishes/plates with overnight incubation at 37 °C for polymerization.

### 4.3. Fluorescent Labeling of Actin

To investigate the filopodia formation using confocal microscopy (see Section 4.7), PANC-1 cells were fixed using 4% PFA, followed by permeabilization (0.1 % Triton-X, Sigma), blocking with 1% bovine serum albumin (BSA, Sigma), and staining with rhodamine-conjugated phalloidin (Invitrogen, Waltham, MA, USA) and DAPI (Invitrogen). To quantify the actin dynamics using TIRF microscopy (see below), PANC-1 cells were transfected with LifeAct-GFP plasmid (Addgene, Watertown, MA, USA) using Lipofectamine 2000 (Invitrogen) as per the protocol.

### 4.4. Immunofluorescence (IF) on Fixed Cells.

PANC-1 cells were fixed with 4% paraformaldehyde (PFA) for 16 min at room temperature. Blocking was performed with 10% BSA for 1.5 h at room temperature. IF was performed using mAb-hERG1-Alexa488 (final concentration of 5 μg/mg protein), Ts2/16 (Biolegend, San Diego, CA, USA, final concentration of 5 μg/mg protein), anti-paxillin antibody (Biolegend, final concentration 5 μg/mg protein). Incubation and revealing steps were performed as in [39].

### 4.5. Cell Migration Assay

The PSCs were hypoxically activated for not more than 18 h in serum-free DMEM media. In parallel, 12 cm^2^ T-flasks were coated with the DM cocktail and incubated at 37 °C for polymerization. One milliliter of matrix cocktail was used to coat six T-flasks. One-hundred thousand PANC-1 cells were resuspended in serum-free media, seeded on the coating and allowed to spread for 1–2 h. The PANC-1 cell migration was stimulated by replacing serum-free medium with conditioned medium of hypoxically activated PSCs (Concept 400, Jouan, Milan, Italy). When K_V_11.1 inhibition was necessary, cells were simultaneously treated with the K_V_11.1 blocker E4031 (Sigma) at a final concentration of 40 µM, used at a concentration (40 µM) proven to be necessary to block K_V_11.1 currents in a rich, protein containing medium [27]. Time-lapse images were acquired using a phase contrast microscope for 4 h in 10 min intervals, as reported earlier [28]. For the analysis of cell migration tracks of individual cells were analyzed. Cell motility was quantified as the movement of the cell center. We determined the speed of individual cells as a mean of each interval during the course of the experiment and the translocation as the net distance covered during a period of 4 h.

### 4.6. Immunoprecipitation and Immunoblotting

All procedures were carried out at 4 °C. Protein were extracted in cold protein extraction buffer (1× cell lysis buffer). For immunoprecipitation, total lysates (1.5 mg) were subjected to a preclearing step by incubating them with Protein A/G Plus-Agarose for 2 h at 4 °C. Co-immunoprecipitation of β1 integrin and K_V_11.1 lysates was performed with mAb-β1. Western blotting was performed on immunoprecipitates and total lysates (input) from the same sample with polyclonal antibodies against K_V_11.1 or β1. Densitometric analysis was performed using ImageJ software (ImageJ 1.38, U.S. National Institutes of Health, Bethesda, MD, USA) on two different scans, after background subtraction, from at least three different experiments. When quantifying variations in K_V_11.1–β1 integrin interactions, the signal for the co-immunoprecipitated protein was first divided by the signal of the protein used for immunoprecipitation. The resulting value is indicated as “K_V_11.1/β1 integrin complex”.

### 4.7. Confocal Microscopy

The fluorescently labelled cells were imaged under a Nikon Eclipse TE2000-U (Nikon, Tokyo, Japan) confocal microscope as described in [38]. The 512 × 512 pixels (195 nm/pixel) images were captured using a 60×/1.4 water immersion objective (Nikon), after sample excitation at 642 nm. The filopodia detection was achieved using custom made MATLAB (MathWorks) scripts based on the method described in [29]. Briefly, a rolling ball filter was applied to the actin signal, which was then shrunk to remove noise, dilated, and skeletonized. To analyze only filopodia, actin filaments within 2 μm of the cell border were taken into account. The (linear) density of filopodia was calculated as the total number of filopodia normalized by the cell perimeter length.

### 4.8. Total Internal Reflection Fluorescence (TIRF) Microscopy

LifeAct-GFP-transfected PANC-1 cells were imaged using an inverted microscope (Axiovert 200; Zeiss, Oberkochen, Germany), equipped with digital camera (Visitron, Puchheim, Germany), TIRF slider (TILL Photonics) and a 100×/1.45 oil immersion objective (Zeiss), after sample excitation at 488 nm. The image acquisition was controlled by the MetaVue software and image series were acquired at 1 Hz for a duration of 2 min. To quantify the actin dynamics, the time series of 1024 × 1024 pixels (60 nm/pixel) images were analyzed using the spatio-temporal image correlation spectroscopy [28]. For each cell, the perimeter was detected and overlapping regions of interest (ROIs) of 32 × 32 pixels (centered around each pixel belonging to the cell perimeter) were analyzed. For each ROI, frames t apart in time were then spatially correlated to obtain 2D Gaussian-like spatio-temporal correlation functions (CFs). By means of a non-linear least squares 2D Gaussian fitting, we obtained the temporal evolution of the CF peak position (up to *t* = 10 s). If flow is present in the ROI, the displacement of the peak over time is directly related to the velocity (i.e., xp = −vxt and yp = −vyt). To determine the minimum velocity that the method is able to detect, we simulated movies (120 frames at 1 Hz) of only diffusing particles (i.e., no flow) in 32 × 32 pixels frames, setting the diffusion coefficient D to 0.25 pixels2/frame. The STICS analysis was been performed on these simulated data and the resulting detected velocity was vmin = 5 ± 2 nm/s. All the obtained velocities were then cleaned according to the threshold and used to build distributions of actin polymerization speed. The growing and retraction were calculated as velocities pointing outwardly and inwardly of the cell perimeters, respectively.

### 4.9. Intracellular Calcium Imaging by Fura2

The intracellular free Ca^2+^ concentration was measured ratiometrically using the fluorescent dye Fura2 (final conc. 3 µM). Fura2-AM was mixed in either PSC conditioned medium or serum free DMEM medium and incubated for 30 min at 37 °C. Dye-loading medium was removed by superfusing the cells with prewarmed HEPES-buffered Ringer’s solution (37 °C) of the following composition (122.5 mM NaCl, 5.4 mM KCl, 1.2 mM CaCl_2_, 0.8 mM MgCl_2_, 5.5 mM D-glucose, and 10.0 mM HEPES (pH 7.4)). Excitation wavelength alternated between 340 and 380 nm, and the fluorescence emission was recorded at 500 nm. Images were acquired in 10 s intervals. Monochromator, camera and data acquisition were controlled by Metaflour software (Visitron Systems, Puchheim, Germany). After a ~10 min control period without superfusion of Ringer’s solution, the hERG blocker E4031 was added and the [Ca^2+^]_i_ was measured for another 10 min. At the end of each experiment, the measurements were calibrated by applying 2 µM ionomycin (MP Biomedicals, Solon, OH, USA) containing Ringer’s solution with 5 mM EGTA or 5 mM Ca^2+^. The [Ca^2+^]_i_ was calculated as described earlier [34].

### 4.10. Statistics

All *p*-values were determined by a Mann–Whitney test (significant level set to *p* < 0.05), after proving the deviation from normality of each condition using a Kolmogorov–Smirnov test. A two sample ANOVA test (significant level set to *p* < 0.05) was used otherwise. 

## 5. Conclusions

The present study was aimed to shed light on the K^+^ channel-dependent molecular mechanism of regulation of cell mobility and f-actin dynamics, we provide evidence that K_V_11.1 is implicated in sustaining pro-metastatic signals in pancreatic cancer, through a reorganization of f-actin in stress fibers and a modulation of filopodia formation and dynamics.

## Supplementary Materials

The following is available online at http://www.mdpi.com/2072-6694/11/2/135/s1, Figure S1: Actin stress fibers formation in HEK 293 cells on FN, Movie S1: Representative TIRF image sequence (120 images at 1 Hz) of live GFP Life-Act transfected PANC-1 cell onto DM, after Hypo-PSC-CM stimulation.
