# Supplementary material for: The Activity of KV11.1 Potassium Channel Modulates F-Actin Organization During Cell Migration of Pancreatic Ductal Adenocarcinoma Cells
Source: Cancers (Basel). 2019 Jan 23;11(2):135. doi: 10.3390/cancers11020135 (PMC6406627; doi:10.3390/cancers11020135)
Supplement: Supplementary file 1 [file cancers-11-00135-s001.zip › cancers-422291 - supplementary materials/cancers-422291 - supple.-final check.docx]

Supplementary Materials: The Activity of Kv 11.1 Potassium Channel Modulates F-Actin Organization During Cell Migration of Pancreatic Ductal Adenocarcinoma Cells

Sagar Manoli, Stefano Coppola, Claudia Duranti, Matteo Lulli, Lara Magni, Nirmala Kuppalu, Nikolaj Nielsen, Thomas Schmidt, Albrecht Schwab, Andrea Becchetti and Annarosa Arcangeli


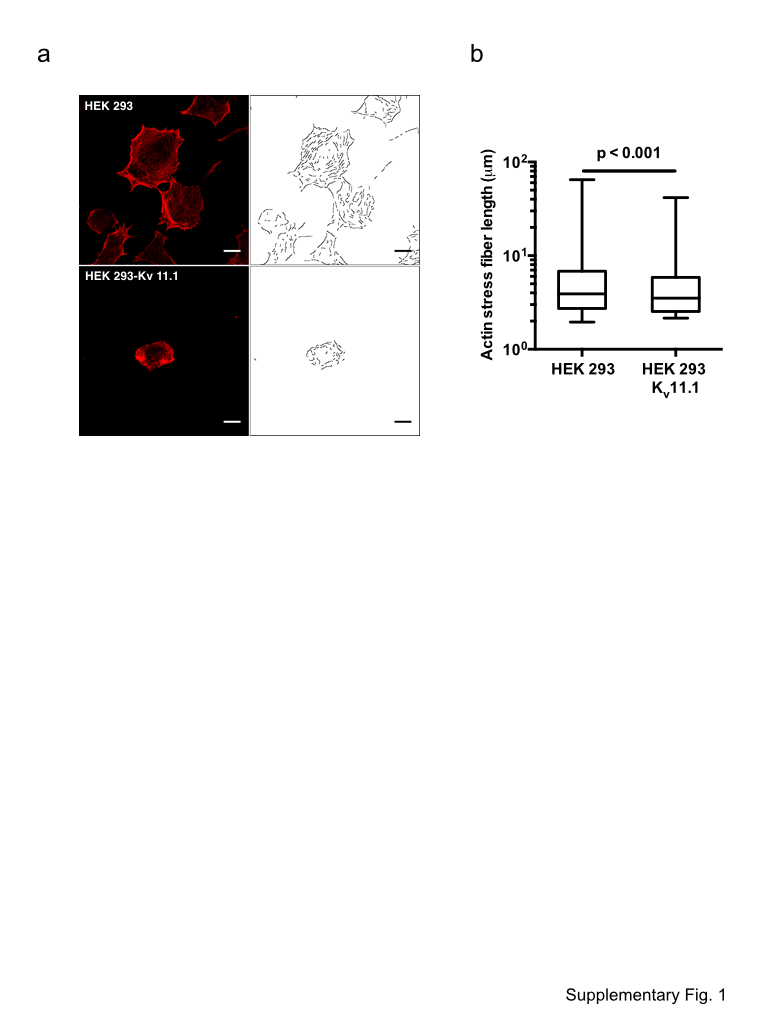


**Figure 1.** Actin stress fibers formation in HEK 293 cells on FN. (**a**) Representative confocal single cell image of HEK 293 and HEK 293-K_V_11.1 cells (**b**) Distribution of actin stress fibers in HEK 293 and HEK 293-K_V_11.1 cells. Scale bar: 10 μm.

**Movie S1**: Representative TIRF image sequence (120 images at 1 Hz) of live GFP Life-Act transfected PANC-1 cell onto DM, after Hypo-hPSC-CM stimulation. Please find at another Supplementary file.

| 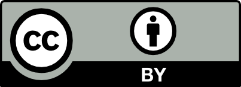 | © 2019 by the authors. Licensee MDPI, Basel, Switzerland. This article is an open access article distributed under the terms and conditions of the Creative Commons Attribution (CC BY) license (http://creativecommons.org/licenses/by/4.0/). |
| --- | --- |
